# Measuring Long-Term Impact Based on Network Centrality: Unraveling Cinematic Citations

**DOI:** 10.1371/journal.pone.0108857

**Published:** 2014-10-08

**Authors:** Andreas Spitz, Emőke-Ágnes Horvát

**Affiliations:** 1 Interdisciplinary Center for Scientific Computing (IWR), University of Heidelberg, Heidelberg, Germany; 2 Northwestern Institute on Complex Systems (NICO), Northwestern University, Evanston, Illinois, United States of America; Katholieke Universiteit Leuven, Belgium

## Abstract

Traditional measures of success for film, such as box-office revenue and critical acclaim, lack the ability to quantify long-lasting impact and depend on factors that are largely external to the craft itself. With the growing number of films that are being created and large-scale data becoming available through crowd-sourced online platforms, an endogenous measure of success that is not reliant on manual appraisal is of increasing importance. In this article we propose such a ranking method based on a combination of centrality indices. We apply the method to a network that contains several types of citations between more than 40,000 international feature films. From this network we derive a list of milestone films, which can be considered to constitute the foundations of cinema. In a comparison to various existing lists of ‘greatest’ films, such as personal favourite lists, voting lists, lists of individual experts, and lists deduced from expert polls, the selection of milestone films is more diverse in terms of genres, actors, and main creators. Our results shed light on the potential of a systematic quantitative investigation based on cinematic influences in identifying the most inspiring creations in world cinema. In a broader perspective, we introduce a novel research question to large-scale citation analysis, one of the most intriguing topics that have been at the forefront of scientific enquiries for the past fifty years and have led to the development of various network analytic methods. In doing so, we transfer widely studied approaches from citation analysis to the the newly emerging field of quantification efforts in the arts. The specific contribution of this paper consists in modelling the multidimensional cinematic references as a growing multiplex network and in developing a methodology for the identification of central films in this network.

## Introduction

Cinema plays a key cultural, creative, and industrial role in today's society. In addition to providing the foundation of an extensive economy [1], it is also widely regarded as the seventh art form [2]. With the increasing accessibility of comprehensive data sets on films that include detailed information about their reception, we are witnessing a rising interest in the quantification of various aspects of cinema even beyond film studies and economics. For instance, several recent studies set out to predict the box office takings of films based on ‘word of mouth’ opinion transmission among the audience [3], critics' endorsements [4], [5], forum discussions and online buzz [6], as well as user activity on Wikipedia [7]. Other lines of research concentrated on predicting Academy Award winners from the set of films that were nominated in a given year [8] or analysed the novelty in film plots based on crowdsourced keywords [9].

### Traditional measures of success in cinema and their shortcomings

As indicated by these examples and given the vast film production accumulated over the past century, our perception of cinema is strongly determined by existing means to evaluate and discriminate between films. Measures that quantify the success of individual films focus primarily on two divergent aspects of success: *1*) commercial appeal by considering economic aspects and distinguishing blockbusters, and *2*) artistic excellence by adopting an aesthetic point of view and favouring experimental art house films [10]. Financial performance of a film is commonly assessed by indicators such as the inflation adjusted box office revenue, audience numbers based on ticket sales, and DVD rentals. Besides the various issues raised by the computation of these measures, such as the proper estimation of inflation adjusted revenue [11], critics and most film scholars denounce this purely economic approach. By focusing on the artistic merits of individual films instead, they promote recognition in form of established awards, festival presence, and professional critical acclaim. As a result, their suggestions usually distinguish ‘difficult’ films with high aesthetic and intellectual level [12], often revealing more about an eccentric canon than about the highlights of the film heritage. Despite the vivid discussions concerning these wide-spread measures of market success and creative excellence [13], none of them are able to offer a quantitative account of a production's importance within film history or the role that a film plays in shaping cinematic paradigms. This key aspect of success is expressed in the inspiration that a film provides to others, for instance due to its innovative style, creativity, and ingenious story-telling [14], even decades after the initial release. Efforts to quantify this long-term impact, which is indicated by the inspiration of subsequent films, are still missing.

### The film citation network

In this article, we propose a novel approach that measures long-term impact in cinema through the analysis of cinematic references. The underlying idea is that when a newly made film enters the body of existing films, it defines itself in relation to the existing films. Given the filmmakers' predilection towards referencing other works in their own films, a tangible way of incorporating the relation to inspiring previous work is through *citation*. In the context of films, citations can take on a wealth of forms and serve diverse artistic and narrative purposes. A production can for instance quote parts of the dialogue or music of an older film, directly feature a short sequence, or use a representative element as a prop. A pertinent example is the James Bond-film entitled ‘Skyfall’ (2012), which is the 23rd instalment of the fifty-year-old spy-story series. The film includes the Aston Martin car in tribute to the older Bond-film ‘Goldfinger’ (1964) and the quartermaster ironically mentions that the exploding ball point pen used as a high-tech gadget in ‘GoldenEye’ (1995) has become out-dated in the meantime. In addition to several references to previous Bond-films, ‘Skyfall’ adheres to various conventions used in action films. For example, the scene in which the main antagonist character arrives in a helicopter accompanied by loud music is reminiscent of the iconic scene from ‘Apocalypse Now’ (1979), while the battle scene on top a moving train is a classic staple of westerns and a popular ingredient of action films. Possible motivations behind such citations are the expression of an attitude towards previous films (like tribute/homage or parody) and the narrative and visual expansion of the world of a film. Either way, citations indicate which films influenced the creation of a certain production and the analysis of citation patterns enables reasoning about the so-called *milestones* of film history.

Despite the lack of research on film citations at a large-scale (for a basic approach see Wasserman et al. [15]), much attention has been given to citations that arise in a variety of different contexts. The most extensively studied area is that of scientific citations, whose quantitative analysis dates back to the appearance of large citation databases and is marked by the pioneering work of Garfield, who introduced the impact factor [16], and Derek de Solla Price who studied the preferential attachment mechanisms among scientific papers [17]. For a comprehensive review of these fundamental endeavours see the chapter entitled “Citation Analysis” in the book of Egghe and Rousseau [18]. The contemporary theoretical studies are rooted in work done by Newman [19] and Barabási et al. [20]. Subsequent papers investigated the dynamics of scientific activity, the emergence of citation patterns, as well as the importance of specific contributions, journals, and scientists [21]–[26]. See Radicchi et al. [27] for a summary of recent advances. Going beyond the familiar setting of scientific publications, there are further interesting systems in which citations play a crucial role, albeit less widely studied. One of them are the so-called legal citations documenting decisions written by judges, which cite one another to establish precedent (see Fowler and Jeon for instance [28]). Another area are patent citations, which arise when patent applications cite other, closely related patents to establish their originality and distinction from previous inventions [29]. For an example studying the time evolution of the United States patent citations see Csárdi et al. [30].

**Figure 1:**
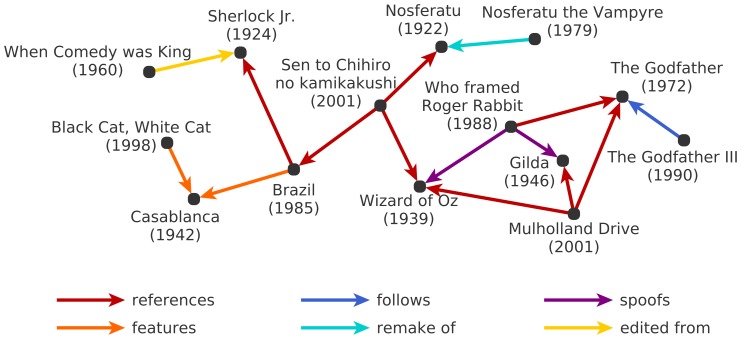
Excerpt from the film citation network extracted from the Internet Movie Database (IMDb). The network contains six different types of citations: The reference is the most common and generic type of citation. In the broadest sense, it can refer to visual or narrative elements that are incorporated into a newer film. For example, the main character in ‘Mulholland Drive’ (2001) consciously assumes the name Rita after seeing the poster of the film ‘Gilda’ (1946), starring Rita Hayworth. A film is said to feature another work if it shows a characteristic sequence from it. For instance, ‘Black Cat, White Cat’ (1998) features ‘Casablanca’ (1942) when its main character watches the ending scene of the latter on TV. Films in a series follow each other. The ‘Godfather II’ (1974) is an example of both sequel of (it picks up the story of the original film) and prequel to (narrates the antecedents) the ‘Godfather’ (1972). A remake is a newer version of an old film, such as ‘Nosferatu the Vampyre’ (1979), which is a stylistic retake on the silent film ‘Nosferatu’ (1922). The spoof relation indicates the ironic imitation of a film, such as the leading character of ‘Gilda’ (1946) that served as an inspiration for the animated and exaggerated character Jessica in ‘Who Framed Roger Rabbit’ (1988). Finally, archive footage from the burlesque ‘Sherlock Jr.’ (1924) is edited into the documentary ‘When Comedy Was King’ (1960), thus exemplifying the most straightforward form of film citation: the direct inclusion of long sequences of the original film.

Since papers, legal decisions, and patents that reference each other can be conceptualized as a growing network, all of these articles employ techniques that have also been used in the study of complex networks and apply them to the task of gaining insight into citation patterns. In line with this approach, we model the body of films that have been released up to date as a network whose nodes are the films and whose edges correspond to citations (see Figure 1). In the following, we refer to this as the *film citation network*. Unlike the previously mentioned networks of citations, this network includes multiple distinct types of references and thus requires novel methodological developments. The formation of this network is governed by the preferences and beliefs of the filmmaker community and it can thus be assumed that the members of this community generate the references (i.e. the edges of the network) with only minor influence from external factors. Therefore, the network contains information about which films are considered by professional insiders to be inspiring works. In addition to compiling such a network, we investigate its structure, develop a framework for the quantification of impact on its basis, identify a list of milestone films through a combination of centrality indices and finally compare this list with diverse selections of ‘great films’.

## Materials and Methods

### The data

We extracted a large trace of this steadily evolving network from the Internet Movie Database (IMDb) [31]. A list of individual files and their sources can be found in Table S1. For technical details on the data preparation step see the Text S1. IMDb has been an approved playground of network research ever since the early papers on small worlds [32], [33] and on scale-free networks [34], [35]. While these studies analysed the collaboration network of actors, subsequent endeavours extended this to additional information that is available on IMDb such as genre, director, producer, and studio [36], plot keywords [9], but also user rating data, budget, and box office revenue [15]. In the latter work the subset of yet another detail present on IMDb is used in an aggregated form, which are the citations between films. This citation information is continuously expanded collaboratively by millions of users and includes records of six types of citations between over 40,000 international feature films going all the way back to the beginning of cinema. Although the categorization of the types of citations in the data does not adhere to any scholarly classification, it indicates relations of varying strength and nature between the films. As suggested by the examples shown in Figure 1, the citation types range from very subtle references and spoofs to explicitly featured sequences of a previous film or even the partial reuse of material by editing them into the new film. While these citation types indicate an acknowledged source of inspiration, films in a series are connected more directly by the follows and remake relations, suggesting that they also share common aspects in terms of story, cast, and/or crew. After the removal of artefacts, the network can be represented as a directed acyclic graph. In the following, we refer to the smaller networks that are generated by these six types of citations as subnetworks, while we call the network that contains all six different types of citations the aggregated network.

Basic statistics of the networks deduced from these data are shown in Table 1. All subnetworks constructed from the single citation types are extremely sparse and consist of several weakly connected components. Due to the nature of the citations, there exist no strongly connected components. The components vary in size and diameter, depending on the subnetwork in question. The references relation is conceptually most diverse and thus the most prevalent type of citation, forming much longer chains of citations than any other single type, as indicated by the high diameter. The next largest subnetworks, namely features and follows, have only half the size of the references subnetwork. The fewest films are involved in the spoofs and edited from citation types and their subnetworks are roughly twice as dense as the rest. A large percentage of the films in the references, features, and spoofs subnetworks are condensed in a single, large weakly connected component which results in larger diameters. We also observe a qualitative difference in the nature of the follows and remake of subnetworks when compared to the rest, as they consist of very small connected components with small diameter. The aggregated network, which contains all six citation types, is clearly the sparsest. It has a much higher number of components than the references subnetwork, yet only a slightly larger diameter, suggesting that many of the small components in the follows and remake of subnetworks are not connected through references relations.

**Table 1:** Statistics for the subnetworks corresponding to the individual citation types and the aggregated network containing all six types.

Citation type	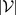	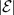	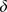	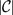	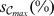	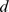
references	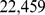	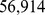	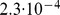	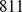	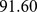	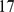
features	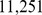	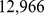	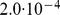	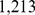	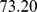	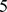
spoofs	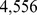	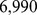	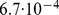	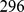	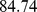	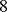
edited from	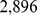	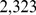	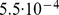	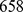	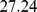	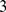
follows	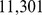	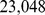	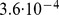	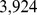	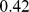	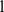
remake of	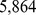	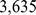	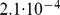	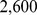	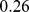	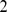
aggregated	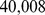	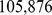	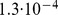	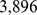	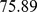	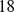

Number of nodes 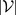, number of edges 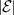, undirected density 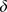, number of weakly connected components 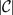, percentage of nodes in the largest weakly connected component 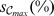, and the directed diameter 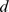 of the network. Note that we give only the undirected density, as there are no reciprocal edges in the network. The directed diameter is computed as the longest directed path in any component of the network. Distances *between* components are disregarded, since all networks contain several clusters and would otherwise have infinite diameter.

**Figure 2:**
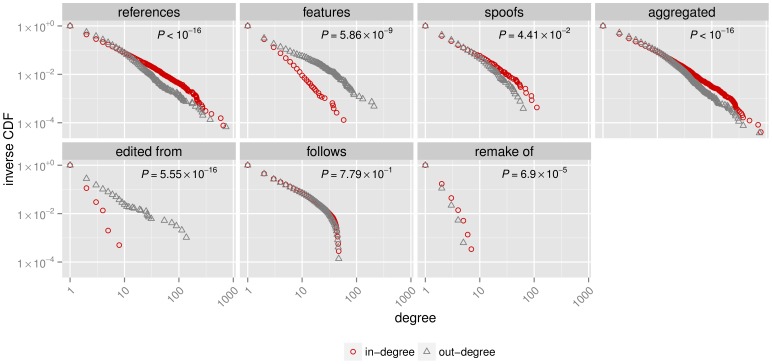
The inverse of the cumulative distribution function for the in- and out-degrees. Plots are shown for the subnetworks constructed from the different citation types and the aggregated network. The *P*-values that result from a Kolmogorov-Smirnov test confirm for all but the follows subnetwork the significant difference between the in- and out-degree distributions.

As shown in Figure 2, the individual subnetworks of the different citation types are very heterogeneous: the in-degree distributions are heavily skewed, indicating the presence of a few hubs that are cited by a large number of films. This is also the case for the number of other films any given film cites, as suggested by the out-degree distributions. We check whether the in- and out-degrees originate from the same underlying distribution using a two-sample Kolmogorov-Smirnov test and find evidence for a significant difference between the two degree distributions for all but the follows subnetwork. The two subnetworks that visibly deviate from the common pattern of a long-tail distribution are those of the follows and remake of citation types. The most likely cause for this is the transitive nature of these two citations. For example, the 1990 film ‘Godfather III’ is a sequel to ‘Godfather II’ released in 1974, which followed the original ‘Godfather’ that was released in 1972. It it obvious that ‘Godfather III’ then also follows ‘The Godfather’. A similar case can be made for the remake of relation, where every remake of a remake is also a remake of the original. Also interesting is that the inverse of the cumulative in-degree distribution function decays faster than that of the out-degree in the case of the features and edited from subnetworks. The higher number of films with a large out-degree requires the presence of several small-degree nodes whose in-degree is larger than their out-degree. This is indicative of the fact that unlike the references or spoofs relation, there is a limit to the footage that can be used in a normal theatrical release, although there exist some documentaries about cinema itself that consist almost solely of such footage.

To further compare the degree distributions, in Table 2 we show the assortativity coefficients of the individual networks, i.e. the Pearson correlation coefficient of degrees between nodes that are connected by an edge [37]. We also include directed assortativities [38], where the direction of the edge is considered and the degrees are divided into in- and out-degrees. Consequently, there are four different types of directed assortativities: 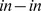, 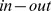, 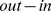 and 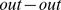. In both cases, larger, positive values mean that nodes tend to be connected to other nodes of similar degree (assortative), while negative values indicate differing degrees of connected nodes (disassortative). We observe that there are two distinct classes of citations with regard to the undirected assortativity. The follows and remake of types are assortative, while the other subnetworks and the aggregated network are slightly disassortative. The most likely explanation for this is the large number of very small components in these two subnetworks. Turning to the directed assortativities, we find more detailed similarities between the subnetworks. Most prominently, the references subnetwork shows no assortativity or disassortativity of any kind and appears to dominate the aggregated network in this regard. The features and edited from subnetworks are very similar to each other, which is intuitive as both types of citation essentially represent original footage of one film that is used in another. In these two networks we observe a high 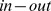 assortativity, indicating that footage of films is either used seldomly in few other films (such as a single scene of an original film that is shown in a sequel), or more frequently in films that use footage from many different sources (such as documentaries). The 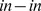 and 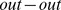 scores are disassortative however, indicating that films that contain footage of other films are less likely to be featured themselves, a distinction which is not true for the references and spoofs subnetworks. The follows and remake of subnetworks are again quite similar, with assortative 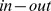 and 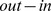 characteristics. The notable difference are the 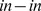 and 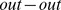 assortativity scores, which indicate that there often exist sequels to films that are sequels themselves, while films that are remakes are less likely to be the basis of another remake.

**Table 2:** Assortativities for the subnetworks corresponding to the individual citation types and the aggregated network containing all six types.

Citation type	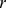	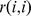	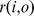	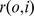	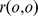
references	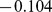	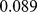	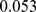	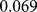	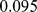
features	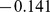	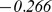	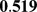	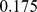	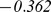
spoofs	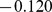	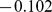	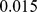	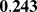	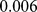
edited from	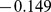	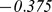	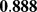	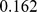	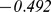
follows	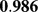	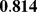	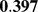	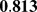	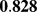
remake of	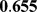	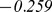	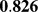	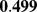	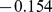
aggregated	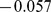	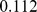	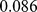	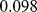	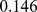

Assortativity of degree 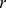 under the assumption that edges in the subnetworks are undirected, as well as the assortativities for directed edges: correlation of the in-degree of source nodes with the out-degree of target nodes 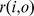, etc. For ease of reading, assortativities above a value of 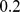 are bold, those below a value of 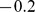 are highlighted in italics.

If we consider the multiplex degree of nodes, i.e. the number of subnetworks a film belongs to, we observe that the subnetworks are fairly separated. Of all films, 68.9% belong only to a single subnetwork, 20.5% can be found on the border between two subnetworks, 7.3% take part in three different types of citations, 2.4% connect four subnetworks and less than 0.9% of all films can be found in five or all six subnetworks. To investigate this further, we calculate node- and edge overlaps between the different subnetworks in the following.

**Figure 3:**
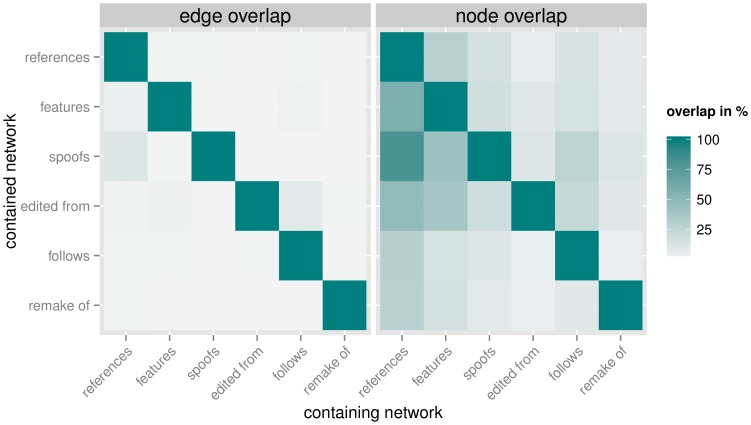
Parallel edges and shared nodes between the subnetworks constructed from the different citation types. Both the edge and node overlap are defined as the percentage of edges/nodes from the network indicated on the y-axis that are also present in the network specified on the x-axis.

Figure 3 shows the overlap between the networks spanned by the different edge types. The overlap is quantified by the percentage of edges from one network (shown on the vertical axis) that are present in the other network as well (horizonal axis). The edge overlap between the six edge types is minimal (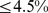) for all combinations, with the exception of the spoofs subnetwork, of which 11.4% are contained in the references subnetwork and the 7.7% of edited from edges that are contained in the follows subnetwork. This is unsurprising, as it is easy to include a reference to a film in a spoof, and sequels often contain material of the original film in a series, such as the 1980 sequel to the 1960 classic ‘Psycho’ that uses the iconic shower scene of the original as its opening scene. The node overlap is more pronounced, especially due to the size of the references subnetwork, which contains 81.1% of the films from the spoofs subnetwork and 58.0% of the films in the features subnetwork. It is reasonable to assume that films that are interesting enough to warrant a production-intensive spoofs or features citation are also prominent enough to be referenced. The overlaps between nodes outside of the references subnetwork are generally smaller (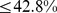). The moderate to negligible overall overlap between the different networks suggests that measures that combine multiple types of citations are more promising in a network-analytic context than those that are limited to just a single type of citation, as they increase the reach of the method, without simply aggregating and treating conceptually different forms of citation the same.

As a summary of this exploration of networks, we find that the distinct nature of the citation types is reflected in the overall structure of the subnetworks, yet many of these characteristics are lost in the complete aggregation of all citation types. In the following, we therefore combine subnetworks belonging to multiple citations only where this is a sensible approach and do not work with a single, aggregated network.

### Centrality of nodes in growing multiplex networks

The film citation network has two important characteristics: *1*) a non-negligible temporal aspect due to the time-ordering of the films given by their release years, and *2*) a multiplex nature, meaning that the qualitatively distinct types of citations require a network model that incorporates different types of edges (corresponding to the various types of citations) between the same set of nodes (the films). While considerable effort has recently been devoted to the analysis of temporal [39]–[43] and multiplex networks [44]–[48], centrality indices [49] that quantify different notions of importance in such networks are just starting to appear (for some examples see References [50]–[52]). In their most common form, the scientific, legal, and patent citation networks that have been analysed record one dimension pertaining to a single aspect of referencing. Film citations on the other hand can be of various types ranging from obvious to rather subtle. The data set at hand comprises multiple orthogonal dimensions of citation in the film setting and thereby introduces a non-trivial multidimensionality that inherently distinguishes the network of film citation from previously studied citation networks. We are aware of only a single previous work on such film citations at a large-scale [15], which reduces the wealth in reference types by aggregating them to one generic citation type. The concept of multiplexity is not uncommon however, and there exist several other systems of this multiplex type. For instance, new companies entering a dynamical market initiate links through which they exchange knowledge or distribute goods. Users of online social networking platforms can be in a wealth of ephemeral relations with e.g. friends, family or colleagues. Another example are transportation systems that offer diverse means of transportation in countries with a booming development. Centrality concepts that consider both the diverse aspects contained in the relations and the fast pace at which they appear, are still lacking yet increasingly needed. The seminal paper of Padgett and Ansell on the structure and interplay between the small marriage, economic, and partonage networks of the Medicean political party in Florentine Renaissance [53] introduces fundamental notions of combined centrality based on different subnetworks. Inspired by this work, we develop a ranking method that is sensitive to the multidimensional nature of edges (and also takes into account the time-ordering of nodes), which is therefore suited for the identification of central nodes in a growing multiplex network. We demonstrate the viability of the approach on the film citation network.

### Identifying milestone films

Our framework for the identification of milestones based on the film citation network relies on a number of adapted centrality indices that quantify different aspects of cinematic influence (see Table 3). The more basic indices are derived from the number of received citations as quantified by the degree centrality. Our first weighted version of the degree centrality accounts for the propensity for citation of the citing films. This assures that a citation has less strength if a film is only one among many that are cited and its gain in centrality is thus diminished due to being shared. Accordingly, the *weighted out-degree centrality* distributes the inspiration equally among the cited films. The second centrality index is a temporal extension of the degree centrality, termed here *temporal degree centrality*. It measures the total time difference between a film and those citing it, thereby emphasizing the importance of the time span between release and the individual citations, since a film that is cited years after its initial release is likely to be more successful in the long term than a film that is only cited while it is still fresh in memory. To take into account the changing trends in film history, the *influence time centrality* is introduced as a further index. It considers the time frame during which the individual film was cited by others. All indices we introduced so far rely on the concept of increased importance through the frequency of direct citations and thus, at least in a first approximation, they can be meaningfully applied to the subnetwork that contains the related citation types features, references, spoofs, and edited from.

**Table 3:** Centrality indices that take into account the time-ordering of the nodes.

Name	Formula	Edge types/subnetwork
weighted out-degree centrality	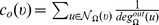	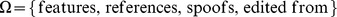
temporal degree centrality	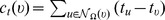	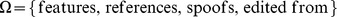
influence time centrality	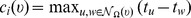	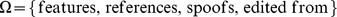
subtree centrality	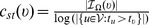	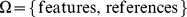
start centrality	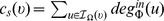	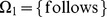 , 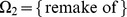
		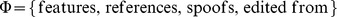
propagation centrality	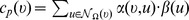	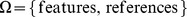
	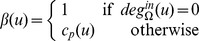	
	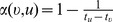	

Individual indices are applied to different subnetworks of the multiplex film citation network. 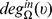 and 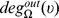 denote the in- and out-degree of node 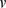 in the subnetwork generated by edges of a type that is contained in the set 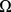, 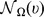 denotes the neighbour set of node 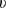 in the same subnetwork, 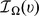 denotes the set of nodes from which 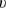 can be reached in the 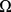 subnetwork, while 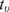 denotes the time stamp associated with node 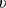. See the Text S1 for further details about the used notation.

As indicated by various examples, indirect effects play an important role in the spreading of inspiration throughout film history. For instance, the German expressionist cinema of the 1920s shaped the visual style and atmosphere of the film noir crime dramas produced in Hollywood in the 1940s and 1950s. Extensions of the latter, produced worldwide since 1960, are referred to as the neo-noir films, which in turn show distinctive thematic and visual elements that are reminiscent of the expressionist style—relations that are often not indicated by direct citations. This motivates the inclusion of path-based indices, which have the additional advantage of mitigating potential noise in the examined large-scale dataset. Based on the subnetwork that is composed of the features and references citation types, the corresponding *subtree centrality* counts the number of films that directly or indirectly cite a given film and normalizes this value by the total number of films in the data set that were released at a later point in time. The *start centrality* counts the number of films, which cite sequels or remakes of the first film in a series. Here, the underlying concept is that films are rewarded for starting highly influential series. We compute this type of centrality separately for both the follows and the remake of subnetwork. In this context, the counted citations are restricted to the remaining types (i.e. features, references, spoofs, and edited from).

Finally, given the assumption that the citation network contains information about the dissemination of narrative and visual solutions in cinema, it is natural to study the spread of these cinematic developments on the network in terms of a diffusion process. Thus, our last centrality index accounts for the propagation of influence gained through citations by taking into consideration the time difference between the cited and citing film (*propagation centrality*). This centrality index follows an approach that is similar to the Katz centrality and Page Rank [49] by assigning an initial amount of influence to films, which is then propagated along the edges of the network. The propagation factor increases as the time span between the release years of the two corresponding films grows larger. Due to the directed acyclic nature of the network, the index can be defined recursively and computed efficiently.

Taken together, these indices incorporate a broad selection of intuitions about what constitutes milestone films based on the temporal citation patterns that can be found in the film citation network. To handle the multiplexity of the network, we combine related edge types for the individual indices where appropriate. The indices address different aspects of centrality (cf. Figure S1). For instance, a highly referenced film can be important without having a sequel, i.e. despite admitting a low rank based on start centrality. To generate the final ranking, we combine the results of the individual indices by using a discounted cumulative rank as follows. We obtain the rank 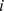 of a given film 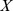 by averaging its ranks 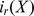 in the diverse rankings 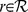, 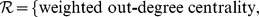 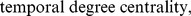 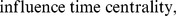 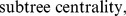 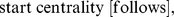 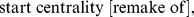 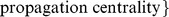. To avoid introducing additional assumptions about the importance of the different centrality indices, we give equal weight to the ranks deduced from them when computing the average. However, to account for the fact that films can be important overall despite being assigned a low rank by a few of the indices, we let the ranks of films *within* a given ranking decrease sublinearly by a factor of 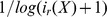. Accordingly, the overall rank of a given film 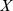 becomes: 
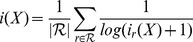
where 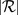 denotes the number of used indices. The resulting highest-ranked films are listed in Figure 2.

## Results

**Figure 4:**
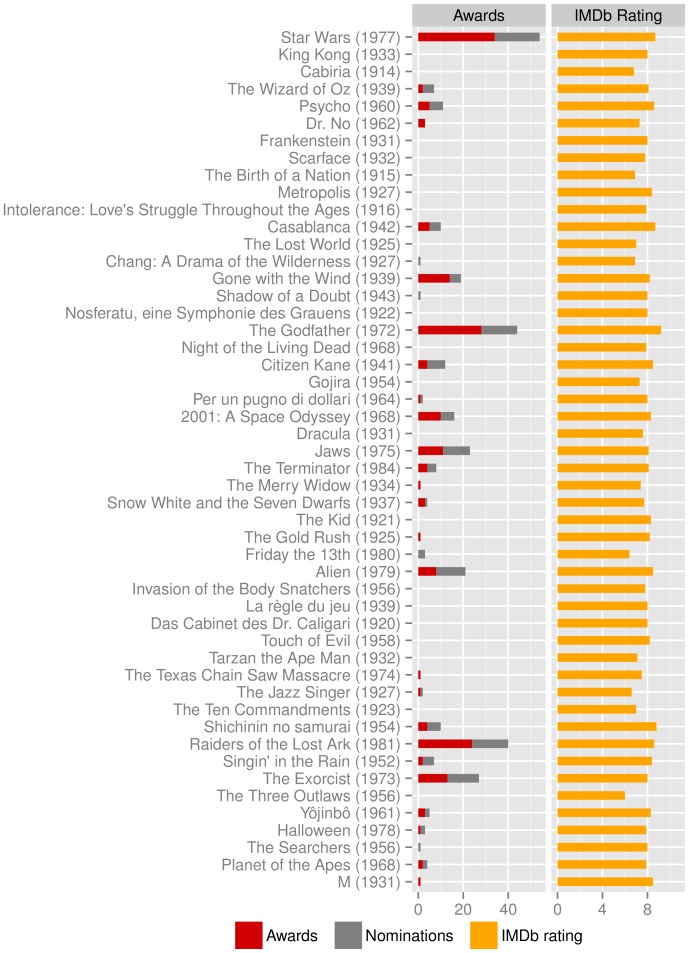
The impact of the 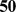 highest-ranked films we extracted from the film citation network as expressed by the number of awards and nominations received within two years of their appearance, as well as their average rating on IMDb. The films are sorted from top to bottom by decreasing ranking according to the film citation network. Ratings are based on a 10 star system with 10 indicating the highest possible rating. Decimal values are possible due to the averaging of individual user ratings by IMDb.

In a first exploratory step, we investigate how the milestone films deduced from the film citation network are assessed by popular traditional measures such as award wins and nominations as well as the average rating given to the film by users of IMDb (individual source files are listed in Table S1). As shown in Figure 4, the majority of milestone films did not receive awards at the time of their release. Notable exceptions are the films of the New Hollywood era from the ‘70s and the beginning of the ‘80s. These were considered blockbusters and their success on the award scene was a part of their marketing strategy. On the other hand, IMDb ratings for the top 50 films range from 6.0 to 9.2 and, with only one exception, the ratings of all milestone films are above the average rating of 6.1. A comparison of our ranking to a ranking based purely on the IMDb user ratings indicates no direct correlation: a Spearman coefficient yields a score of 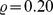. Even if we limit our selection to just the top 10 milestones, the selection still contains two films with a rating of less than 7.0. Fitting a normal distribution to the ratings is not feasible (c.f. Figure S2), but an empirical one-tailed P value of 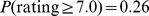 reveals that over a quarter of all films in the data set have a rating of at least 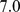. Therefore, a high IMDb rating is neither necessary nor sufficient to obtain a high ranking based on the film citation network. These results indicate that none of these factors (i.e. the number of obtained awards and the ratings) can be conclusively associated with the long-term impact quantified by film citations.

### Comparison with other rankings

**Figure 5:**
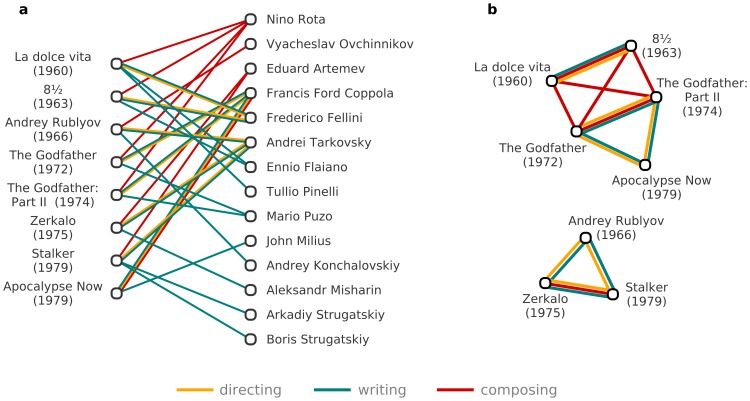
Transforming a film top list into a network based on shared film attributes. (a) Subgraph of the film–attribute bipartite graph containing film–director, film–writer, and film–composer relations. (b) Exemplary subgraphs of the corresponding multiplex film network.

To obtain insight into the characteristics of our selection of milestone films, we compare the top films according to our ranking with a representative set of typical rankings (so-called *top lists*) of diverse origin: personal favourite lists compiled by four randomly chosen individual IMDb users, the top 100 entries of IMDb's regular voters, lists released by two experts in the field (Tim Dirks and Roger Ebert), and curated lists resulting from larger polls of experts, such as those provided by the British Film Institute and the American Film Institute. Since the ranking criteria of the films in these top lists are usually not known and the exact order is thus largely subjective, we transform all lists into networks, whose structure we can then compare to find distinguishing features. We construct networks from film lists by using film attributes, such as the main creators (i.e. the director, writer, and composer), the cast (i.e. the actors), and the genre of the individual films. We represent the films and their associated attributes as a multiplex bipartite graph, in which the films constitute one of the node sets, while the attributes form the other node set (see Figure 5a). We reduce the film–attribute bipartite graph to a multiplex network of films based on shared attributes. Specifically, we link each pair of films that have at least one common creator, actor, and genre, respectively. In doing so, we acknowledge the similarity between the two linked films. (Individual files from the IMDb and sources of the top lists used in this section are listed in Table S1.)

We divide the obtained multiplex network into one that consists of films connected due to shared actor(s) or genre(s) and another that consists of three types of edges indicating shared director(s), writer(s), or composer(s) (see Figure 5b). The networks then contain up to two and three edges of different type between the same pair of films. To quantify the amount of parallel edges, we compute an *edge overlap* for the shared actor and genre network (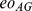) and one for the shared creator network (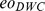) according to the following formulas: 
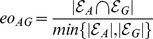
and 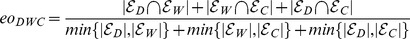
where 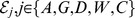 denotes the edge set of shared actor (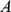), genre (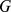), director (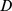), writer (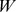), and composer (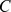). The actor–genre overlap 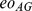 normalizes the number of edges in the intersection of two edge sets 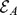 and 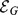 by the number of edges in the smaller set. The director–writer–composer overlap 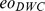 extends this same concept to the case of three edge sets. The range of both functions is the interval 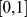, where a value of 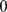 signifies that there are no overlapping edges and a value of 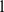 indicates that all edges in the smaller set(s) are overlapping with edges of the larger set(s).

The overlaps quantify two different aspects of diversity within each of the lists: *1*) In a network where films are connected by a distinct type of edge if they share a director, composer, or writer, a diverse list should result in minimal overlap, as films that share much of their driving artistic force can be expected to be similar in terms of plot and style. *2*) Typecasting of actors, i.e. contracting the same actors for similar roles in similar films, is a sign of lacking diversity. Thus, we expect a varied list to show minimal overlap between actors, especially within the same genre.

**Figure 6:**
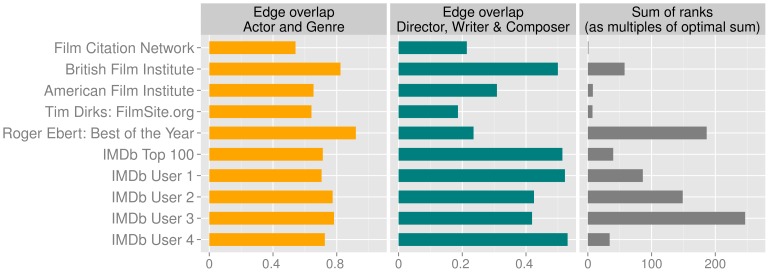
Comparison of various top lists: the personal favourite lists of four randomly chosen IMDb users; the IMDb Top 100, which is based on the votes of its regular users; two lists compiled by the film experts Roger Ebert and Tim Dirks; the lists of the American and British Film Institutes; and our list deduced from the film citation network. (left and center) Overlap between the edges in the multiplex film networks, indicating the ratio of shared attributes. (right) Rankings of all films in the given top lists as compared to the ranking based on the film citation network. For each such list, the ranks of all it's films according to the film citation network ranking are summed and divided by the sum of optimal ranks (i.e. 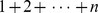 for a list of 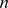 films). The average result for a randomly selected list of 100 films from the data set is 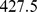 (not shown).

For each top list we compute the edge overlaps 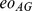 and 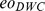 as defined above. As shown in Figure 6, the general trend is that typecasting is more frequent in the considered lists than recurring collaboration between creators. The network deduced from the milestone films (top row) exhibits the least amount of overlap overall, i.e. it has the smallest overlap in the actor and genre network and the second smallest overlap in the shared creators network. This indicates that the list of milestone films provides a more diverse selection than any of the lists used for comparison.

If a list of films was to be selected from the body of all available films uniformly at random, it would also be very likely to consist of films with little overlap between actors and genres as well as creators. We therefore show that while it is more diverse, our list is significantly more similar in terms of node overlap to existing top lists created by renowned institutions and critics than to a purely random list of films. To this end, we preprocess the top lists used for comparison by removing the few instances of direct-to-video releases and TV-shows they contain, bringing them in line with the IMDb data set. We then assign to each film within the individual lists the rank it obtained in our list. We compute the sum of these ranks and divide it by the minimal sum that could be achieved in such a way: 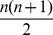 where 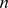 is the number of films in the given list. For our own list, this then results in a value of 1 by construction. A low score for a list indicates similarity to our list, while higher values denote that on average, our approach ranked this list's films poorly. The results of this comparison are shown in Figure 6 (right). Accordingly, our list is more similar to all tested lists than it is to a random list, which would have a similarity to our list of 427.5 on average. Our list is most similar to the lists of the American Film Institute and Tim Dirks, followed by the British Film Institute, the IMDb Top 100 ranking and one of the IMDb user lists (IMDb User 4). The remaining lists of users (IMDb User 1–3) score significantly worse. The list of Roger Ebert is also very dissimilar, which is to be expected since it is constructed on the premise of one film per year. Since Roger Ebert was not active during the first half of the 20th century, many highly ranked milestones are excluded by default from his list. We conclude that by basing a list on the film citation network, it is possible to effectively identify films that critics are likely to consider to be important milestones as well.

## Discussion

The addressed concept of long-term impact has long been subject of a more general art historical discussion. Based on the assumption that the creation of art is a reflective activity, Baxandell suggests to describe an artwork's relation to its circumstances by following two guidelines [54]: *1*) The effect of inspiration through other works should not be treated on the level of artists, but on the level of the artworks themselves, meaning that a given work of art is influenced by other works of art rather than an artist being influenced by its peers. *2*) To track the connection between a given work of art and those previously created, a notion of causality should be included. This can be achieved by considering how, during the process of creation, the artist makes an intentional selection from an array of resources in the history of his craft or even other areas. Thus, instead of stating that artwork X *influenced* artwork Y, it should rather be indicated that Y actively referenced X. This approach also enables differentiating between several ways in which such references may occur. While it was not Baxandell's intention to introduce networks to the analysis of art, it is clear in retrospect that the approach he described can best be represented on a large-scale on the basis of directed, multiplex networks.

Along the lines of these two suggestions, we investigate impact in cinema as manifested through citations. As opposed to in-depth studies of the influence of specific directors or films, our large-scale analysis relies on a vast user-compiled data instead of just personal experience and thereby covers a considerably larger body of films than could be studied previously in this respect. However, the use of such data also introduces certain limitations: being a website in English, the IMDb is predominantly focused on English-speaking films (c.f. Figure S3 and [15]). Due to the nature in which data is accumulated by its users, citations in films that receive more attention from the public are more likely to be detected. This introduces a bias, since foreign films are included in the network mostly when they inspire other films, not when they are being inspired.

A different kind of noise that is undoubtedly contained in the network are false edges, i.e. references that were erroneously identified by users of the IMDb. While the users can in general not be regarded as film experts, there is evidence that for tasks that do not require rigorous training, there is little difference between data obtained from crowdsourcing and expert opinion [55]. The resulting noise is random rather than systematic, as it is caused by variations in preference between individual users, not flaws in design or malicious activity. Heterogeneous networks, such as the film reference network, are well known to be robust against noise, as long as the noise is random instead of targeted at the most well-connected nodes [35], [56]. Like many network-analytic approaches, our method is robust to incidental changes in the network structure due to the size and structure of the network itself. We are able to identify the same set of milestone films with only slight fluctuations in the ranking, even when using earlier versions of the data set. The fact that the selection of top films remains unaltered indicates that the developed combination of adjusted centrality indices provides a robust result. Alterations in the set of top ranked films are to be expected only after significant increases in the number of citations.

Even though the data used in this article does not enable tracking intermedial influences for films, i.e. sources from other art forms such as literature or music, these too play a key role in shaping cinema. It would be a simple task to adapt and extend our approach to such a holistic analysis, provided that the corresponding data is collected.

The film citation network analysed in this article strongly differs from the citation networks that are extensively studied at the moment. Scientific and legal citations are formally documented in the publications and decisions themselves. Besides the opinion of patentees and their attorneys, patent citations also reflect the references found by patent examiners during consideration of the application. The film citation network however goes one step further in externalizing the instance that identifies the citations entirely. In this case, IMDb's regular voters are those who record the citations as they become apparent to them. Additionally, film citations can range from very explicit to more subtle references and can be linked to diverse elements of the visual and narrative world of the film (e.g. dialogue, details of the set, or parts of the musical score). Thus, due to the way filmmakers reference previous works and based on how they are identified by the multitude of users, film citations are more informal than traditional citation contexts. This informal nature of the edges, the multidimensionality of the relation types and the fast evolution of the network extend the relevance of our modelling formalism and the presented method even beyond the domain of art networks.

## Supporting Information

Figure S1 **Spearman correlation between the different centrality indices.** The correlation is computed based on the films that have non-zero centrality with respect to at least one of the computed indices (i.e. 17,704 films in total). The start centralities as computed from the remake of and follows subnetworks respectively have near-zero correlation values with the rest of the indices, because they involve a different set of films. Although there is a slightly higher correlation between the remaining measures, the plot shows that the different indices quantify complementary aspects of importance in the film citation network.
(TIF)

Figure S2 **Distribution of the ratings in the considered IMDb data set.** The red curve represents a normal fit to the data indicating that this is not a good approximation to the data.
(TIF)

Figure S3 **Histograms showing the release year, genre, and language of the films contained in the cleaned data.** (top) The considered selection of films resemble the increased production over time. The peak in the ‘30s and ‘40s corresponds to the Golden Age of Hollywood. (bottom, left) The most frequent classifications are the generic categories of comedy and drama. The films are associated with multiple genres and often contain dialogue in multiple languages. (bottom, right) Although most films are in English, the main European and Asian film industries are also represented in the data set. The plot is restricted to the 15 most frequent languages.
(TIF)

Table S1 **Overview of the used data sets and their sources.** All data is freely available.
(PDF)

Text S1 **Details about data collection and preprocessing alongside the required network definitions.**
(PDF)
