# Supplementary figures and images for: Measuring Long-Term Impact Based on Network Centrality: Unraveling Cinematic Citations
Source: PLoS One. 2014 Oct 8;9(10):e108857. doi: 10.1371/journal.pone.0108857 (PMC4189979; doi:10.1371/journal.pone.0108857)

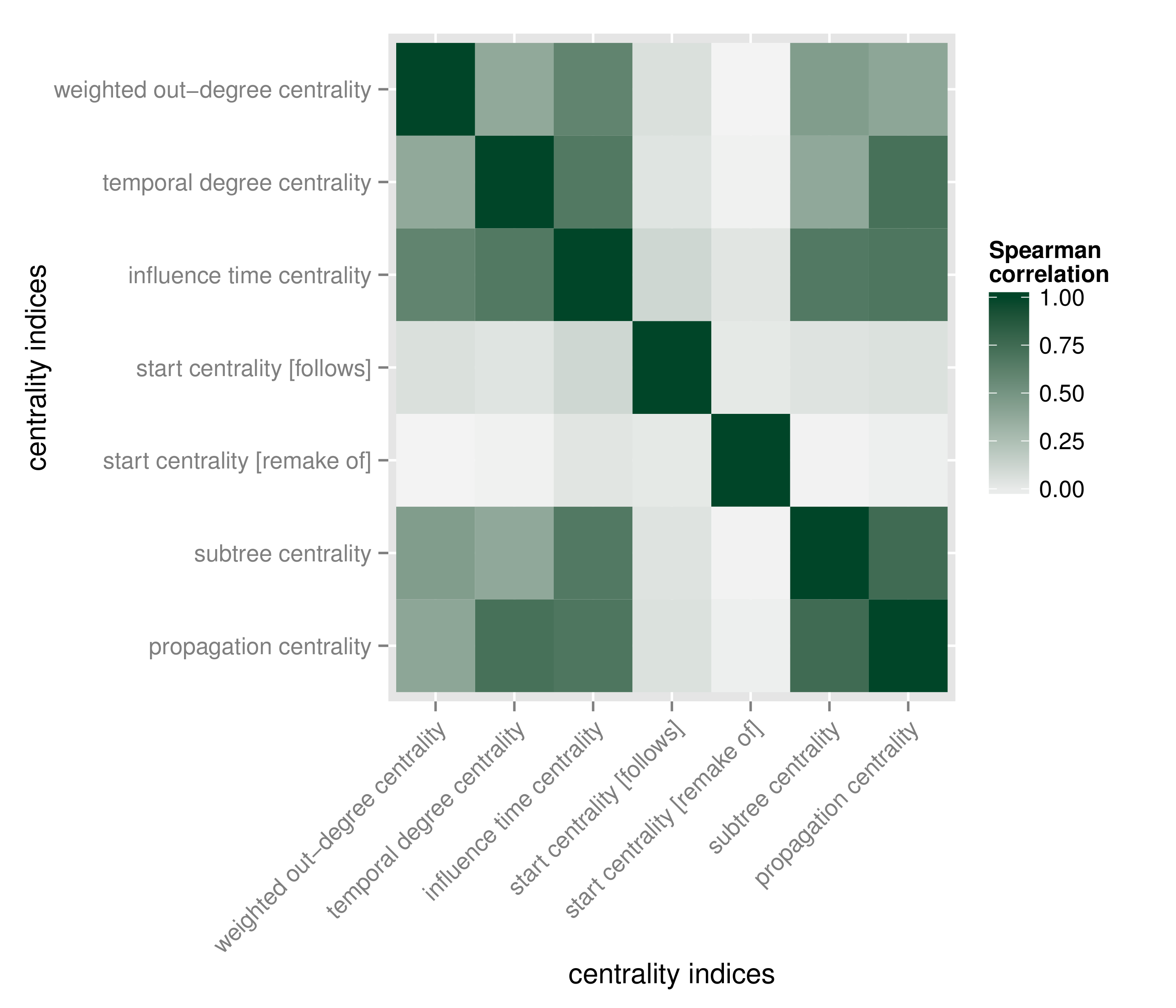

Supplement: Figure S1 — Spearman correlation between the different centrality indices. The correlation is computed based on the films that have non-zero centrality with respect to at least one of the computed indices (i.e. 17,704 films in total). The start centralities as computed from the remake of and follows subnetworks respectively have near-zero correlation values with the rest of the indices, because they involve a different set of films. Although there is a slightly higher correlation between the remaining measures, the plot shows that the different indices quantify complementary aspects of importance in the film citation network. (TIF) [file pone.0108857.s001.tif]

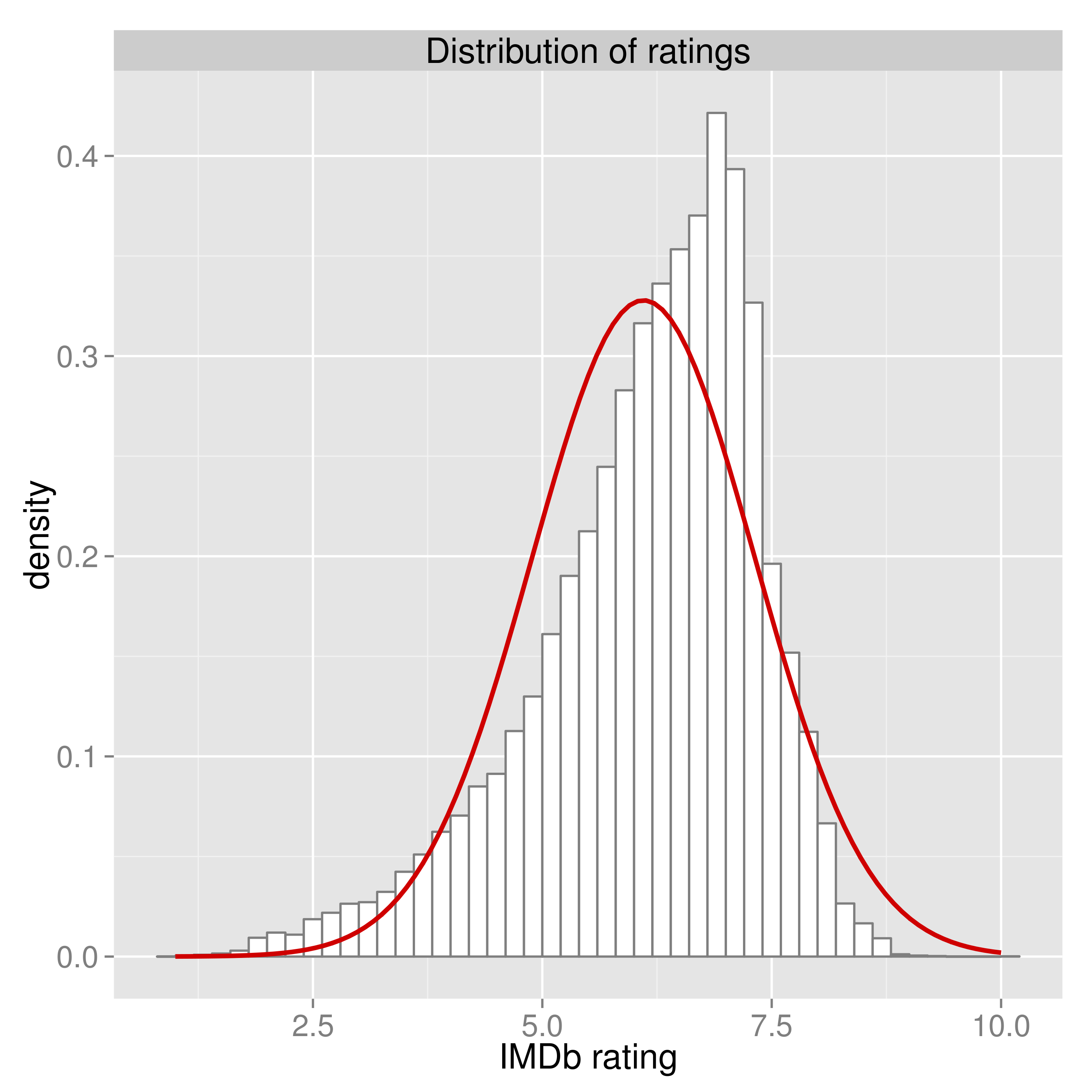

Supplement: Figure S2 — Distribution of the ratings in the considered IMDb data set. The red curve represents a normal fit to the data indicating that this is not a good approximation to the data. (TIF) [file pone.0108857.s002.tif]

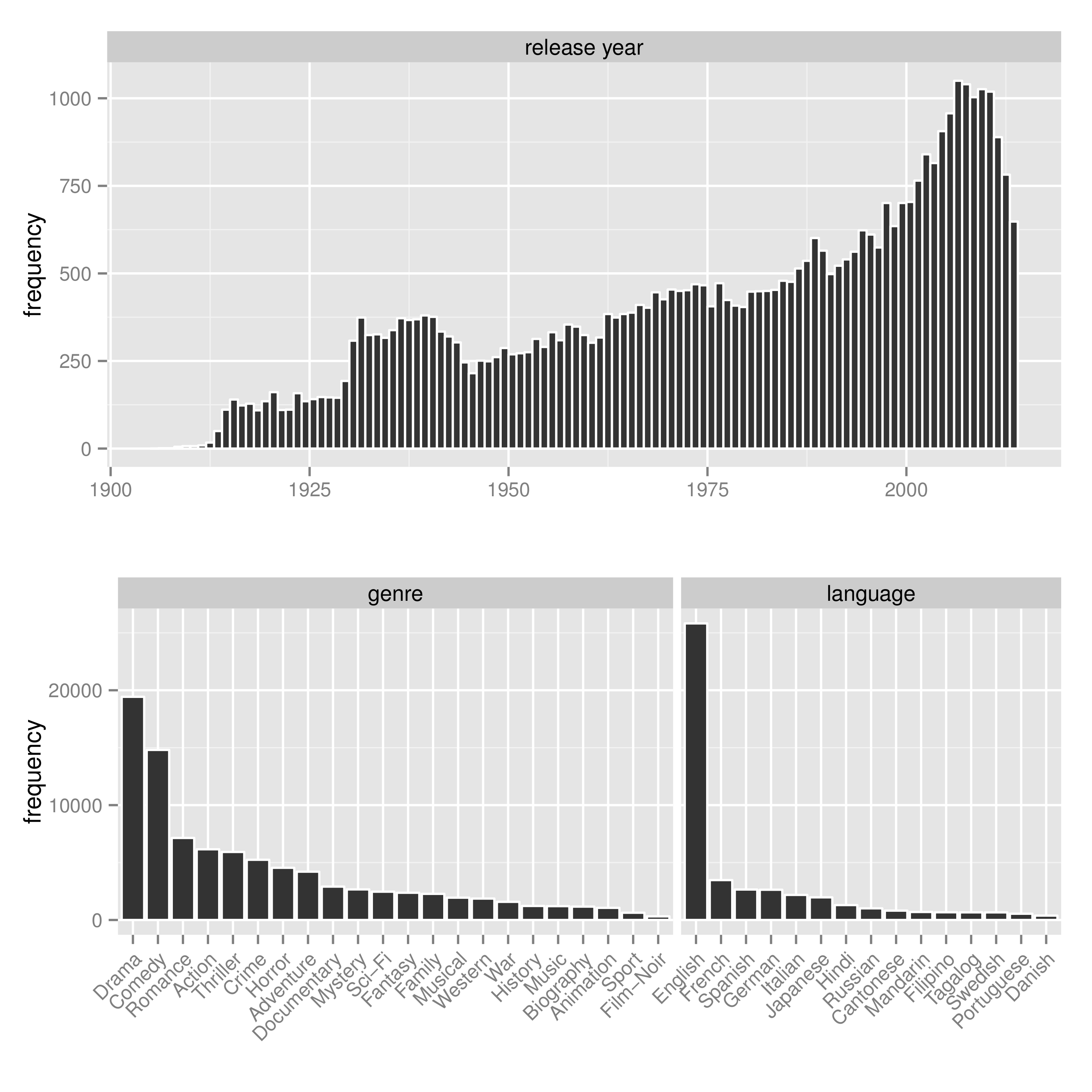

Supplement: Figure S3 — Histograms showing the release year, genre, and language of the films contained in the cleaned data. (top) The considered selection of films resemble the increased production over time. The peak in the ‘30s and ‘40s corresponds to the Golden Age of Hollywood. (bottom, left) The most frequent classifications are the generic categories of comedy and drama. The films are associated with multiple genres and often contain dialogue in multiple languages. (bottom, right) Although most films are in English, the main European and Asian film industries are also represented in the data set. The plot is restricted to the 15 most frequent languages. (TIF) [file pone.0108857.s003.tif]
