# Supplementary material for: Measuring Long-Term Impact Based on Network Centrality: Unraveling Cinematic Citations
Source: PLoS One. 2014 Oct 8;9(10):e108857. doi: 10.1371/journal.pone.0108857 (PMC4189979; doi:10.1371/journal.pone.0108857)
Supplement: Table S1 — Overview of the used data sets and their sources. All data is freely available. (PDF) [file pone.0108857.s004.pdf]

Format: Text files		
Data	Source Name	File Name
Citation information	IMDb	movie-links.list.gz
Average film rating	IMDb	ratings.list.gz
Actors and Actresses	IMDb	actors.list.gz & actresses.list.gz
Film genres	IMDb	genres.list.gz
Directors	IMDb	directors.list.gz
Writers	IMDb	writers.list.gz
Composers	IMDb	composers.list.gz

Format: Scraped HTML files		
Data	Description	URL
Award wins and nominations	Awards page of the individual films on IMDb	e.g. for the film Wings (1927) http://www.imdb.com/title/tt0018578/awards?ref_=tt_ql_4
List of the American Film Institute	Based on more than 1,500 members of the film community who picked the 100 best films of all time from a list of 400 movies nominated by the institute	http://www.infoplease.com/ipea/A0760906.html#ixzz2UV18M8fP
List of the British Film Institute	Based on the votes of 846 critics, programmers, academics and distributors	http://www.bfi.org.uk/news/50-greatest-films-all-time
List of Roger Ebert	Ebert compiled "best of the year" movie lists beginning in 1967	http://www.buzzfeed.com/ellievhall/roger-eberts-top-films-of-his-career
List of Tim Dirks	Written and edited by Tim Dirks on filmsite.org	http://www.filmsite.org/momentsindx.html#100greats
IMDb Top 100	The first 100 entries from the IMDb Top 250 as voted by regular users	http://www.imdb.com/chart/top
List of IMDb User 1	Personal top movies list by *Dennis van Straalen* created 11 July 2011	http://www.imdb.com/list/ls005513983/
List of IMDb User 2	Movies 2 Watch by *bwalt22* created 31 May 2013	http://www.imdb.com/list/ls053771856/
List of IMDb User 3	Phenomenal films by *meghanmaxwell94* created 31 May 2013	http://www.imdb.com/list/ls053777147/
List of IMDb User 4	Personal Favourites by *WillBP* created 4 February 2012	http://www.imdb.com/list/ls002700805/

Supplementary Table S1: Overview of the used data sets and their sources. All data is freely available. The URLs were last accessed on 2014 Sep 8.
