# Supplementary material for: Measuring Long-Term Impact Based on Network Centrality: Unraveling Cinematic Citations
Source: PLoS One. 2014 Oct 8;9(10):e108857. doi: 10.1371/journal.pone.0108857 (PMC4189979; doi:10.1371/journal.pone.0108857)
Supplement: Text S1 — Details about data collection and preprocessing alongside the required network definitions. (PDF) [file pone.0108857.s005.pdf]

Supplementary Text S1

Measuring long-term impact based on network centrality: unraveling cinematic citations
by Andreas Spitz and Emőke-Ágnes Horvát

Data collection and preprocessing

As of November 22, 2013 the IMDb contained citation information on 220, 268 distinct titles in the data file `movie-links.list` available for download [1]. The data set consisted of seven types of citations that can be modelled by reciprocal directed edges (`references`–`referenced in`, `features`–`featured in`, `follows`–`followed by`, `remake of`–`remade as`, `spoofs`–`spoofed by`, `edited from`–`edited into`, `spin off from`–`spin off`) and two types of undirected citations (`version of` and `alternate language version of`). Of these we selected and used those six types of citations that can be related to the propagation of inspiration among the films. The type `spin-off` in particular was removed because it refers to TV-shows only. To avoid redundancy, we introduced a convention by which we reduced all reciprocal edges to single edges, directed from the newer to the older films (in agreement with [2, p. 58]). Next, we eliminated productions that comply with one of the following rules: *1*) productions that have not been released yet ($t > 2013$), but have an IMDb entry, *2*) works in production with unknown release year or future release that have already been cited, for instance as part of the marketing strategy, and *3*) the few instances of citations where an older film cites a newer one, either due to error or delayed release dates. This avoids citation cycles. Our study focuses on feature films and we thus disregarded video releases, video games, TV series, and TV films. After also removing films belonging to the genres *adult*, *game show*, *news*, *reality-tv*, *short*, and *talk show*, we obtained a cleaned data set containing 40, 008 feature films and 105, 876 citations. Table 1 in the main text shows the frequency of the different citation types in this dataset.

Network definitions

In this article, networks are modelled as graphs $G = (\mathcal{V}, \mathcal{E})$ that consist of a set of nodes $\mathcal{V}$ and a set of edges $\mathcal{E}$ that connect the nodes. For directed edges, we write $(v \to w)$ if the edge goes from node v to node w. Multiplex networks contain multiple types of edges between the same set of nodes. Let Ω denote the set of edge types. A multiplex network with edges of types in Ω is then defined as $G_\Omega = (\mathcal{V}, \cup_{\gamma\in\Omega}\mathcal{E}_\gamma)$, where $\mathcal{V}$ denotes the set of nodes and $\mathcal{E}_\gamma$ denotes the set of edges of type $\gamma \in \Omega$. $\mathcal{N}_\Omega(v)$ is the set of neighbours of node v such that $\mathcal{N}_\Omega(v) = \{u \in \mathcal{V} : (u \to v) \in \cup_{\gamma\in\Omega}\mathcal{E}_\gamma\}$. The in-degree of node v is $deg^{in}_\Omega(v) = |\mathcal{N}_\Omega(v)|$; its out-degree is $deg^{out}_\Omega(v) = |\{u \in \mathcal{V} : (v \to u) \in \cup_{\gamma\in\Omega}\mathcal{E}_\gamma\}|$. A path between the nodes v_1 and v_k is defined as $P_\Omega(v_1, v_k) = \{v_1, v_2, \ldots, v_k\}$, i.e. a sequence of nodes such that $v_j \in \mathcal{V}$ and $(v_j \to v_{j+1}) \in \cup_{\gamma\in\Omega}\mathcal{E}_\gamma\, \forall 1 \le j < k$. Then, $\mathcal{I}_\Omega(v) = \{u \in \mathcal{V} : \exists P_\Omega(u, v)\}$ denotes the set of all nodes from which v can be reached by directed paths, and is said to be the in-component of v [3, p. 143–145] except v itself. Each node v is associated with a set of attributes, such as the time stamp t_v, which is equal to the release year of the film represented by node v.

References

1. The Internet Movie Database (IMDb). Alternative interfaces. http://imdb.com/interfaces/.

2. Baxandall M (1985) Patterns of intention: On the historical explanation of pictures. Yale University Press.

3. Newman ME (2010) Networks. An introduction. Oxford.
